# Adsorption characteristics of organics in the effluent of ultra-short SRT wastewater treatment by single-walled, multi-walled, and graphitized multi-walled carbon nanotubes

**DOI:** 10.1038/s41598-018-35374-8

**Published:** 2018-11-22

**Authors:** Yifei Zha, Yuanyuan Wang, Shuzi Liu, Shuai Liu, Yaqiong Yang, Hangcheng Jiang, Yuankai Zhang, Lu Qi, Hongchen Wang

**Affiliations:** School of Environment & Natural Resource, Renmin University of China, No. 59 Zhongguancun Street Haidian District, Beijing, 100872 China

## Abstract

With a conceptual shift in sewage treatment from ‘waste pollution’ to ‘vehicle of resource and energy recovery’ and the further intensification of the energy crisis, the separation and recovery of carbon resources from discharged sewage has gained increasing recent attention in the field of water treatment. The ultra-short Solids Retention Time (SRT) activated sludge process (SRT ≤ 4 d) is highly efficient for separating organic matter and improving the energy recovery rate in wastewater treatment plants, but the effluent quality is relatively poor. If organics in the ultra-short SRT effluent can be reduced further to separate and recover carbon resources, the process may soon replace the traditional activated sludge process. We conducted physical adsorption carbon recovery experiments in an ultra-short SRT (SRT = 2 d) activated sludge system using three carbon nanotubes. Considering that Chemical Oxygen Demand (COD) arises from a mixture of organic compounds, and because humic acid (HA) makes up a large fraction of the effluent and can cause great environmental harm, further experiments were conducted on the adsorption of HA in the effluent COD to three nanotubes. This study proposes a novel method to completely remove organics from the effluent from ultra-short SRT activated sludge processes and reveals nanotube adsorption properties and mechanisms.

## Introduction

Recent years have seen a worldwide conceptual shift in sewage treatment from ‘waste pollution’ to ‘vehicle of resource and energy recovery.’ Organic matter in sewage contains 1.5–1.9 kW·h·m^−3^ of chemical potential, which is nearly 10 times the energy consumption of sewage treatment^1,2^. If this potential source of energy can be used effectively, wastewater treatment plants (WWTP) will not only be self-sufficient in terms of energy use, but may also be able to provide energy to the wider grid, eventually becoming actual energy processing plants. Therefore, the separation and recovery of carbon resources from discharged sewage has gained increasing attention worldwide in the past ten years in the field of water treatment. The ultra-short Solids Retention Time (SRT) activated sludge process (SRT ≤ 4 d) developed from section A of the traditional Absorption Biodegradation (AB) process is highly efficient in separating organic matter from water and improving the sewage plant energy recovery rate. The process it poised to become the leading wastewater treatment technology, but currently produces relatively poor effluent quality, with a Total Chemical Oxygen Demand (TCOD) at around 70 mg·L^−1^. TCOD in the wastewater effluent can be divided into three components, namely, Particle COD (PCOD), CCOD (Colloidal COD), and Soluble COD (SCOD)^3–5^. Although the PCOD and CCOD removal rates are satisfactory, the SCOD content is still high, requiring further reduction of the effluent COD to meet discharge standards. If the organic matter in the ultra-short SRT effluent can be reduced to 50 mg·L^−1^, achieving further separation and recovery of carbon resources, this technology be able to replace traditional activated sludge processes more quickly.

Previous studies have demonstrated that the secondary effluent SCOD from municipal wastewater treatment plants still contains some refractory organics, such as humic acid (HA), fulvic acid (FA), and synthetic organic compounds. When these organic compounds are broken down by microorganisms in the water, dissolved oxygen (DO) is consumed. Therefore, higher levels of organic contaminants cause decreased DO levels, leading to oxygen deficiency in the water and simultaneously producing H_2_S and NH_3_, causing black and odorous water. In addition, HA can pollute the ultrafiltration membrane, greatly increasing the cost of drinking water production^6^. In addition, HA contains functional groups, such as carboxyl, hydroxyl, and amino groups, that have strong adsorption and complexation properties. Thus, HA not only affects the separation of other small molecules in water, but also plays an important role in the enrichment of heavy metals^7^. Toxic compounds in the water enter the food chain and eventually threaten human health, potentially causing liver failure, brain dysfunction, and even cancer^8^. Therefore, the complete separation of dissolved organic matter (DOM), and especially HA, in the effluent is important for public health and environmental safety.

To reduce COD from 70 mg·L^−1^ to 50 mg·L^−1^ in effluent from ultra-short SRT activated sludge process, it is unrealistic to design a new microbial treatment system given that the majority of organic matter is not easily degraded by microorganisms. Physical adsorption is the simplest advanced wastewater treatment method for some degree of DOM removal; adsorption is highly efficient, has little environmental impact, occupies little space, and uses recyclable adsorbents in contrast to chemical treatment methods.

New carbon materials, such as carbon nanotubes (CNTs), based on traditional adsorbents (such as activated carbon and diatomite) have gained attention in synthetic adsorbent research in recent years. The most widely used CNTs are Single-Walled Carbon Nanotubes (SWCNTs) and Multi-Walled Carbon Nanotubes (MWCNTs)^9,10^. SWCNTs involve a single-layer structure, while MWCNTs have a multilayer structure. Therefore, the physical properties of the two differ^11^. The CNT organic matter separation mechanism involves chemical and physical adsorption (van Edward force), including hydrogen bonding and π-π bond^12–14^, which are closely related to the chemical properties of the CNT surface. Graphitized Carbon Nanotubes (GWCNTs) feature a smaller number of oxygen functional groups on the surface than do MWCNTs^15^. Thus, we employed SWCNTs, MWCNTs, and GWCNTs in our experiment to determine the effects of physical properties and oxygen functional groups on organic matter separation processes. Select recent studies have studied the adsorption of organic matter by CNTs, finding that CNTs possess excellent organic matter adsorptive properties^16,17^. However, no research has been conducted on CNT adsorption of effluent COD from the ultra-short SRT activated sludge process. Currently, the DOM-to-CNT adsorption mechanisms remain poorly understood.

We conducted experiments on carbon recovery in an ultra-short SRT (SRT = 2 d) activated sludge system using SWCNTs, MWCNTs, and GWCNTs. First, we conducted an effluent COD adsorption experiment. Because COD arises from a mixture of organic compounds, and because HA makes up a large fraction of the effluent and can cause great environmental harm, further experiments were conducted on the adsorption of HA in the effluent COD to three CNTs. Understanding the adsorption mechanisms of these CNTs will not only provide theoretical support for environmental risk assessments, but also provide ideas and technical background for the application of carbon nanomaterials in carbon resource recovery and utilization.

## Results

### Removal of COD from short SRT wastewater treatment effluent

#### Variations in COD with time in CNT adsorption tests

**Figure 1:**
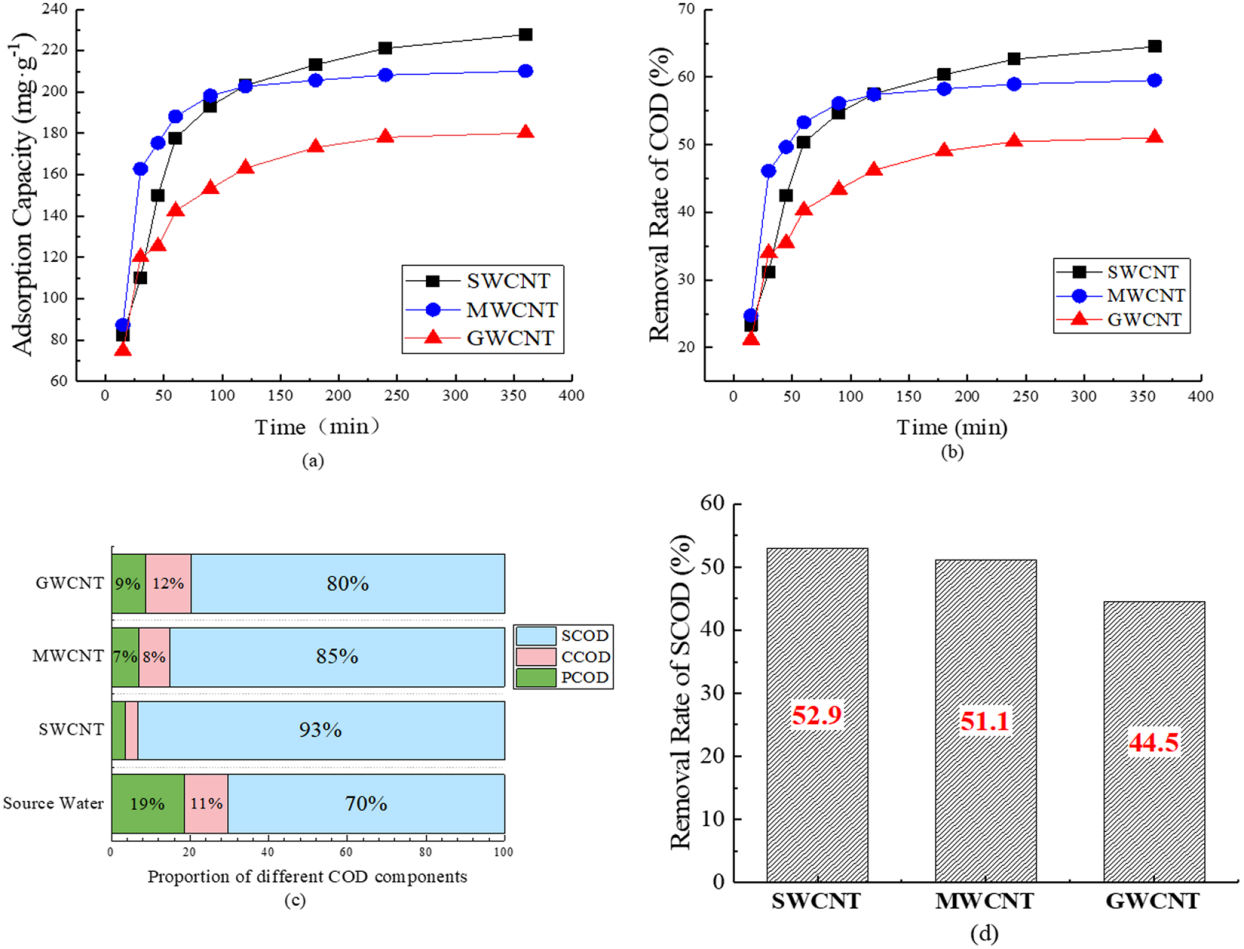
Removal of COD from short SRT wastewater treatment effluent. Variation of the COD adsorption capacities on three CNTs with time (**a**). Variation of removal rate of COD by three CNTs with time (**b**). Proportion of different COD components in source water and effluent from SWCNT, MWCNT, and GWCNT adsorption tests (**c**). Removal rate of SCOD by SWCNT, MWCNT, and GWCNT (**d**). (The concentration of COD is about 70 mg/L. The concentration of CNTs is 200 mg/L. pH = 7. T = 298.15K).

The variations in the adsorption capacity and removal rate of COD by SWCNT, MWCNT, and GWCNT materials are presented in Fig. 1(a,b). The adsorption rate of organic matter was high at first but decreased sharply over time. Adsorption equilibrium was attained at 360 min.

MWCNT showed the highest rate to reach the maximum adsorption capacity of COD at 210.2 mg·g^−1^ among the three CNTs (Fig. 1(a,b)). Nevertheless, SWCNTs achieved the highest COD adsorption capacity (227.8 mg·g^−1^). SWCNT also had the highest TCOD removal rate (64.5%), followed by MWCNT (60.0%). GWCNT featured the lowest COD adsorption capacity, at 180.2 mg · g^−1^, and the lowest removal rate (51.0%).

#### Characterization of organic matter in the water before and after adsorption tests

The distribution of three COD components in source water and effluent from SWCNT, MWCNT, and GWCNT are presented in Fig. 1(c,d) along with the SCOD removal rate. After the adsorption of organics in the source water, PCOD and CCOD together accounted for 6.6%, 15.0%, and 20.2% in effluent from SWCNT, MWCNT, and GWCNT, respectively. SCOD was also removed efficiently by the three CNTs.

**Figure 2:**
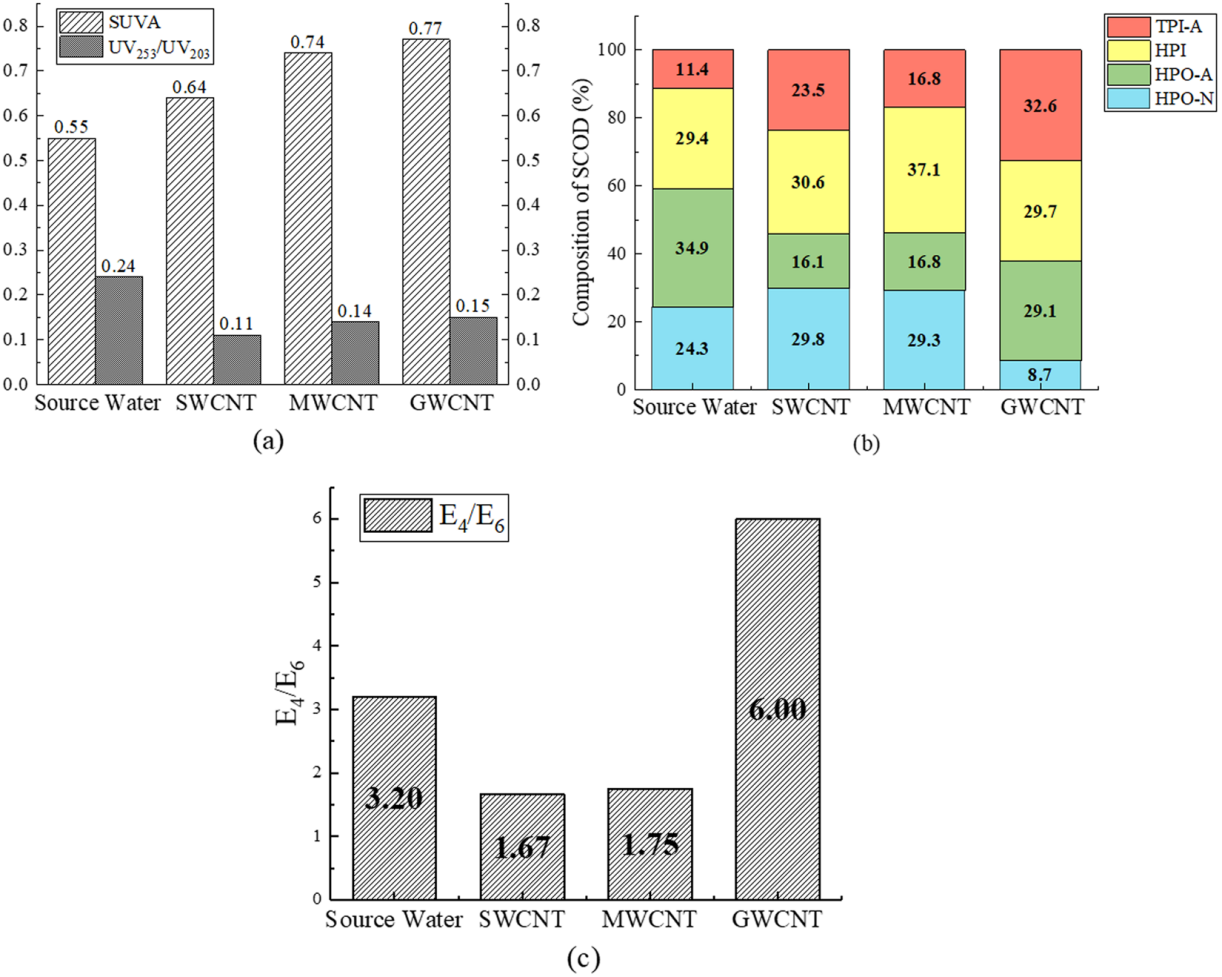
Characterization of organic matter in the water before and after the adsorption tests. SUVA and UV_253_/UV_203_ of source water and effluent from SWCNT, MWCNT, and GWCNT adsorption tests (**a**) HPO‒A, HPO‒N, TPI-A and HPI of source water and effluent from SWCNT, MWCNT, and GWCNT adsorption tests (**b**) E_4_/E_6_ of source water and effluent from SWCNT, MWCNT, and GWCNT adsorption tests (**c**).

Since organic matter in the effluent after CNT adsorption included large SCODs fraction, experiments were conducted to characterize the SCOD in source water and in the effluent of CNTs. Specific UV Absorbance (SUVA, defined as UV254 normalized with respect to DOC) and UV_253_/UV_203_ data are shown in Fig. 2(a). SUVA is an ideal indicator of aromaticity and double-bond structures (such as those found in humic substances) of DOM in the water and closely related to the generation potential of halogens^18^. UV_253_/UV_203_ exhibits illustrates the degree of -OH, -COOH, -C=O, and lipid substitution for aromatic rings. Higher aromatic ring abundance is associated with increased potential for disinfection by-product production^19,20^. SUVA increased from 0.55 in the source water to 0.64, 0.74, and 0.77 after adsorption to SWCNTs, MWCNTs, and GWCNTs, respectively. UV_253_/UV_203_ decreased from 0.24 in the source water to 0.11, 0.14, and 0.15 after adsorption to SWCNTs, MWCNTs, and GWCNTs, respectively, showing the lower degrees of aromatic ring substitution. Figure 2(b) presents HPO-A, HPO-N, TPI-A, and HPI data for the source water and SWCNT, MWCNT, and GWCNT effluents obtained from XAD-8 and XAD-4 resin columns; SCOD in wastewater typically consists of these four components^21,22^. The molecular weight of SCOD influences the biological stability of the water body, reflected by E_4_/E_6_ (Fig. 2(c)). Larger E_4_/E_6_ values indicate higher amounts of low molecular weight organics in the water^23^. E_4_/E_6_ reduced dramatically from 3.20 in source water to 1.67 and 1.75 in the effluent of the adsorption test on SWCNT and MWCNT, respectively, but increased sharply to 6.00 after treatment by GWCNT.

#### Effects of temperature, adsorbent concentration, and pH

The temperature, adsorbent concentration, and pH exert great influence on absorbance, and thus were subject to adsorption tests to explore the mechanisms of CNT organic adsorption.

**Figure 3:**
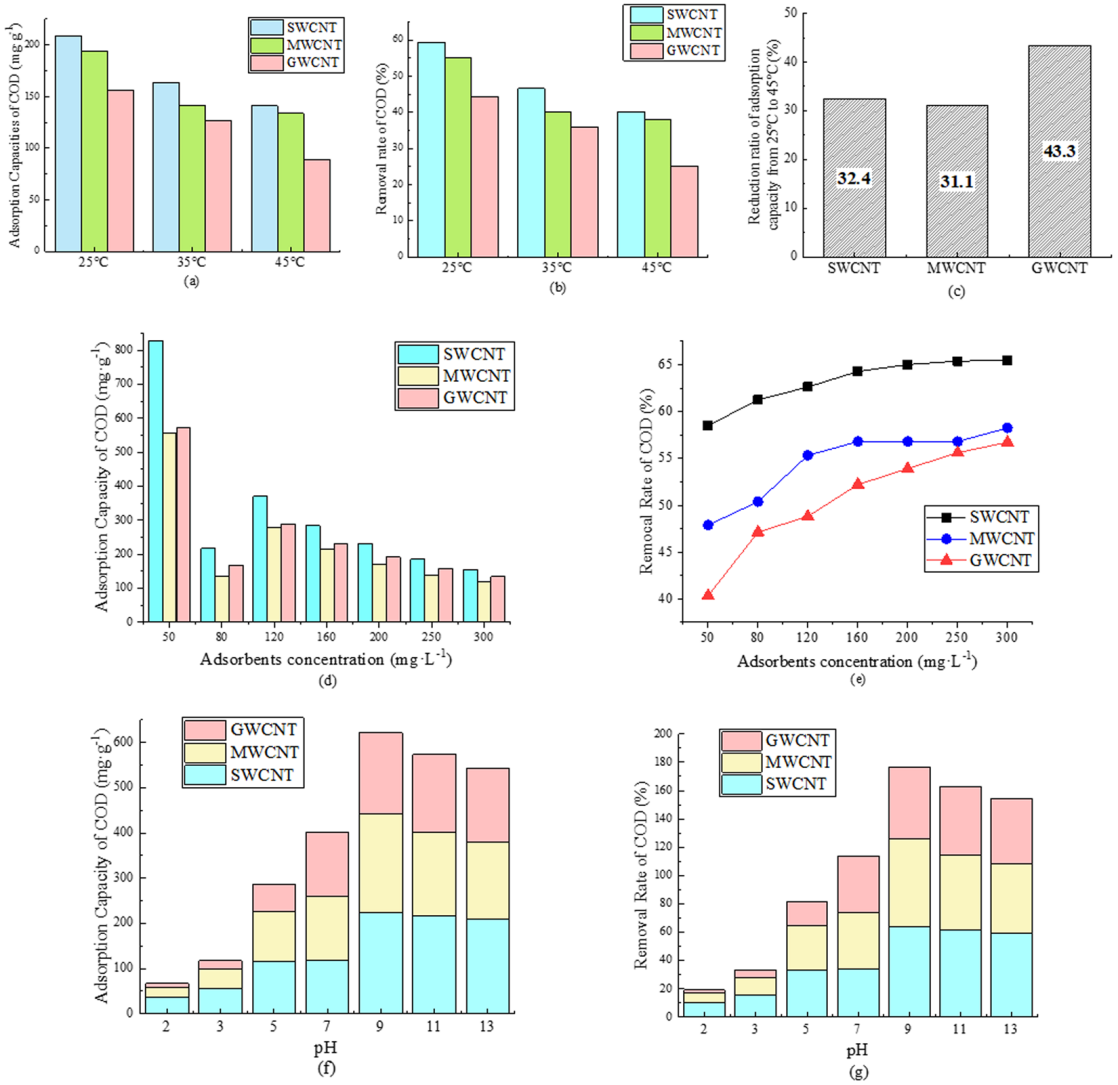
Effects of temperature, adsorbent concentration, and pH. Adsorption capacities of COD of CNTs at 25, 35, and 45 °C (**a**). Removal rate of COD of CNTs at 25, 35, and 45 °C (**b**). Reduction ratio of adsorption capacity under a temperature change from 25 °C to 45 °C (**c**); (The concentration of COD is about 70 mg/L. The concentration of CNTs is 200 mg/L. pH = 7). COD adsorption capacities of CNTs at varying CNT concentrations (**d**). Removal rate of COD of CNTs at varying CNT concentrations (**e**); (The concentration of COD is about 70 mg/L. The concentration of CNTs are 50, 80, 120, 160, 200, 250 and 300 mg/L. pH = 7. T = 298.15K). Adsorption capacities of COD of CNTs at varying pH (**f**). Removal rate of COD of CNTs at varying pH (**g**). (The concentration of COD is about 70 mg/L. The concentration of CNTs is 200 mg/L. pH was controlled at 2, 3, 5, 7, 9, 11, and 13. T = 298.15K).

Adsorption tests for COD in the source water were conducted at 25, 35, and 45 °C to study the effects of temperature; the results are shown in Fig. 3(a,b). As the temperature increased from 25 to 45 °C, the COD adsorption capacities and removal rates by SWCNTs, MWCNTs, and GWCNTs all decreased. The rates of reduction in adsorption capacity are shown in Fig. 3(c) for SWCNTs, MWCNTs, and GWCNTs under an increase in temperature from 25 to 45 °C. The SWCNT and MWCNT adsorption tests were less affected by the temperature, with reduction ratios of 30.0%. In contrast, the reduction in COD adsorption capacity was 43.3% in the GWCNT adsorption tests.

Different concentrations of CNTs (50–300 mg·L^−1^ CNTs) were used in adsorption tests to understand the effect of adsorbent concentrations. In Fig. 3(d,e), the CNT adsorption capacity decreased with increasing adsorbent concentrations. Although the COD removal rates increased at higher CNT concentrations for these three CNTs, the rates of increase became lower.

The solution pH was adjusted from 2 to 13 to study the effect of pH on COD adsorption. The resulting COD adsorption capacities and removal rates are shown in Fig. 3(f,g). Both values changed dramatically with pH; both adsorption capacity and COD removal rate were maximized at pH = 9.

### Removal of typical organic contaminants in the effluent

Because there are different types of organic matter in the source water, exploration of the adsorption mechanism requires the identification of organic matter in the effluent. Dissolved organic matter in natural aquatic environments consists of a complex mixture in which the representative organic compounds are HA and FA^24^. These compounds may both affect the appearance of the aquatic environment and cause animal carcinogenesis through the formation of disinfection by-products, which also threaten human health^8^. In addition, HA contains functional groups, such as carboxyl, phenol, hydroxyl, and amino groups, that have strong adsorption and complexation effects on pollutants. Thus, HA not only affects the separation of other small molecules in water, but also plays an important role in the enrichment of heavy metals^7^. Therefore, it is necessary to study the ability of CNTs to separate HA. We selected HA as a representative of organic matter and performed experiments on adsorption kinetics, isotherm responses, thermodynamics, and the effect of varying pH to understand HA adsorption mechanisms on the three CNTs.

#### Kinetic studies

**Figure 4:**
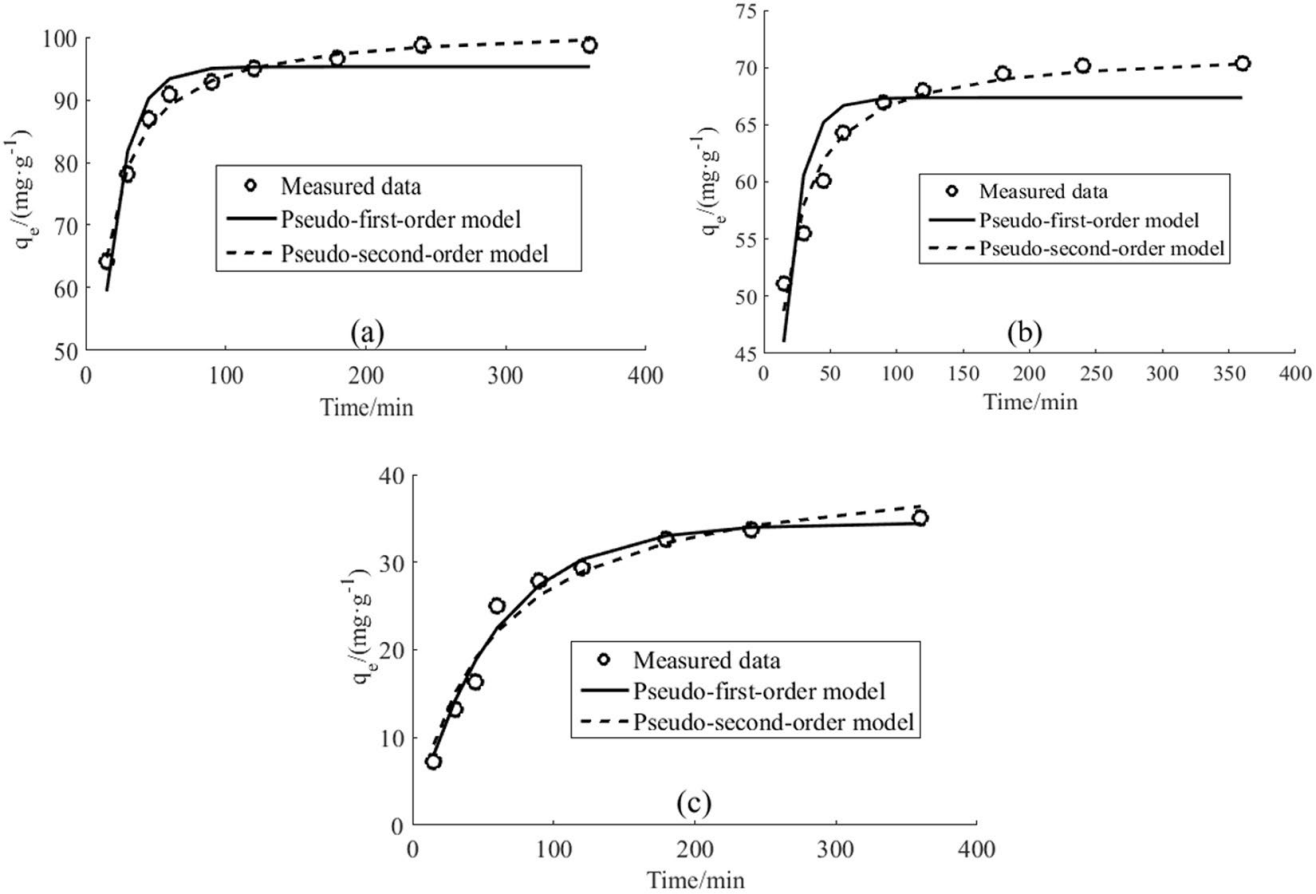
Adsorption kinetics of HA on SWCNT (**a**), MWCNT (**b**), and GWCNT (**c**) (The concentration of HA is 20 mg/L. The concentration of CNTs is 200 mg/L. pH = 7. T = 298.15K).

To determine the adsorption mechanisms and rate control steps of HA adsorption on CNTs, pseudo first- and second-order models and internal diffusion kinetic models were applied, respectively. The results are presented in Fig. 4 and Table 1a. The adsorption capacity of the three CNT types increased with time and adsorption equilibrium was attained at 6 h. Of the three CNTs, SWCNTs had the highest adsorption capacity (98.8 mg·g^−1^), followed by MWCNTs (70.4 mg·g^−1^). GWCNTs featured an adsorption capacity of 35.0 mg·g^−1^, which is 49.7% that of MWCNTs.

**Table 1:** Parameters involved in the adsorption of the HA on CNTs.

a. Parameters for the Lagergren pseudo-first- order models and pseudo-second-order models.									
CNTs	q_e,exp_ (mg·g^−1^)	pseudo first-order model			pseudo second-order model				
		q_e,cal_ (mg·g^−1^)	k_1_ (g·mg·min^−1^)	R^2^	q_e,cal_ (mg·g^−1^)	k_2_ (g·mg·min^−1^)	R^2^		
SWCNT	98.8	95.3	0.0651	0.908	101.9	0.0011	0.992		
MWCNT	76.5	67.4	0.0765	0.691	71.7	0.0020	0.953		
GWCNT	35.0	34.5	0.0176	0.974	41.8	0.0004	0.955		
**b. Parameters for the internal diffusion kinetics model**.									
**CNTs**	**Stage 1**			**Stage 2**			**Stage 3**		
	**K**_**int1**_ **(mg·g** ^**−1**^ **·min** ^**−0.5**^ **)**	**C**_**1**_	**R** ^**2**^	**K**_**int2**_ **(mg·g** ^**−1**^ **·min** ^**−0.5**^ **)**	**C**_**2**_	**R** ^**2**^	**K**_**int3**_ **(mg·g** ^**−1**^ **·min** ^**−0.5**^ **)**	**C**_**3**_	**R** ^**2**^
SWCNT	8.035	33.40	0.997	1.279	80.87	0.995	0.371	92.09	0.669
MWCNT	3.187	38.51	0.992	1.168	55.44	0.975	0.158	67.54	0.789
GWCNT	2.340	0	0.915	1.380	14.45	0.990	0.422	27.00	0.986
**c. The Freundlich model fitting parameters for the adsorption of the HA on CNTs**.									
**CNTs**	**T (K)**	**K**_**F**_ **(mmol** ^**1−n**^ **·L** ^**n**^ **·kg** ^**−1**^ **)**	**n**	**R** ^**2**^					
SWCNT	298.15	7.20	0.919	0.981					
	308.15	3.61	0.935	0.976					
	318.15	3.22	0.763	0.956					
MWCNT	298.15	6.67	0.451	0.991					
	308.15	3.39	0.862	0.977					
	318.15	2.03	0.888	0.990					
GWCNT	298.15	5.58	1.432	0.968					
	308.15	1.47	0.996	0.967					
	318.15	0.12	0.773	0.973					
**d. Thermodynamic parameters of the adsorption of the HA on CNTs**.									
**CNTs**	**T (K)**	**ΔG (KJ·mol** ^**−1**^ **)**	**ΔH (KJ·mol** ^**−1**^ **)**	**ΔS (KJ·mol** ^**−1**^ **·K** ^**−1**^ **)**					
SWCNT	298.15	−14.51	−94.55	−0.27					
	308.15	−11.82							
	318.15	−9.14							
MWCNT	298.15	−14.66	−52.72	−0.13					
	308.15	−13.39							
	318.15	−12.11							
GWCNT	298.15	−13.75	−64.10	−0.17					
	308.15	−12.07							
	318.15	−10.38							

Figure 4 and Table 1a show that the pseudo-second-order model features high correlation coefficients (R² > 0.95) and thus better describes the kinetics of HA adsorption by SWCNTs and MWCNTs. However, for GWCNTs, the pseudo-first-order model had a higher correlation coefficient (R^2^ = 0.974) than did the pseudo-second-order model (R^2^ = 0.955).

**Figure 5:**
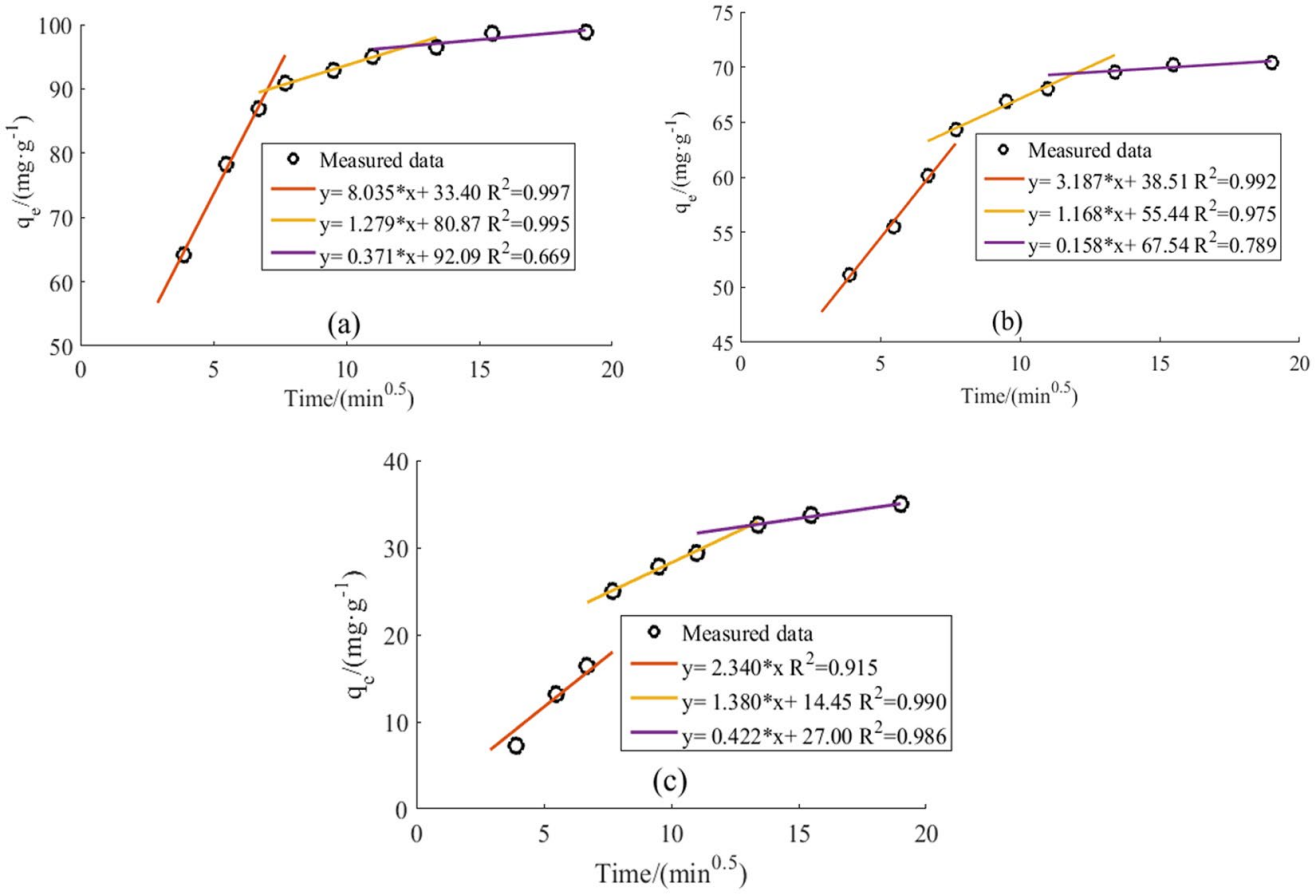
Fitting curve of internal diffusion kinetics of the HA adsorption on SWCNT (**a**), MWCNT (**b**) and GWCNT (**c**).

Diffusion processes, including external diffusion, interface diffusion, and pore diffusion, play key roles in the adsorption process^25^. Pore diffusion is the most important factor affecting adsorption dynamics, and an internal diffusion model can be used to determine the control step in the adsorption process^26^. The CNT adsorption sites generally exist on the outer surfaces and micropores of the carbon nanotubes^27,28^. To better understand HA adsorption and separation processes in CNTs, the experimental data were fitted with the internal diffusion kinetic model, as shown in Fig. 5. The adsorption of HA on CNTs was divided into three continuous stages. In Stage 1 (0–45 min), HA was adsorbed or instantaneously adsorbed on the surface of the CNTs with 0.915 < R^2^ < 0.997. In Stage 2 (45–120 min), CNTs gradually adsorbed HA with 0.990 < R^2^ < 0.995. In Stage 3 (120–360 min), with the gradually decreasing HA concentration, the effect of diffusion became weaker and adsorption equilibrium was eventually reached. The R² of this stage ranged from 0.669 to 0.986. Parameters for the internal diffusion kinetics model are presented in Table 1b.

#### Isotherm and thermodynamic studies

Langmuir and Freundlich(FM) isotherm model were all used for data analysing. Result revealed that the Freundlich model (0.956 < R^2^ < 0.991) provides a better fit than the Langmuir model (0.899 < R^2^ < 0.943) as far as fitting accuracy is concerned. Thus, HA is adsorbed to the multilayer molecules on the CNTs. Details on the Freundlich model results are listed in Table 1c at temperatures of 25, 35, and 45 °C. Thermodynamic parameters of HA adsorption on CNTs are shown in Table 1d.

#### Effect of pH

**Figure 6:**
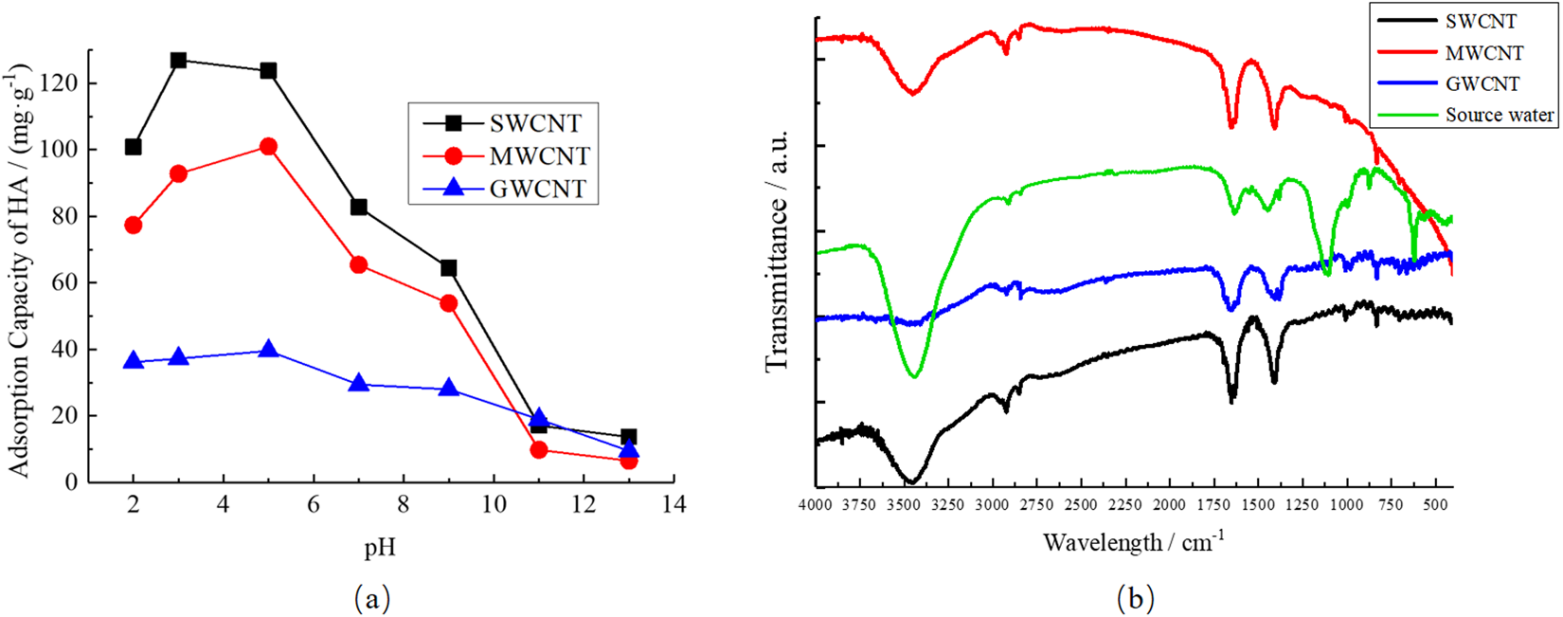
Adsorption capacities of SWCNTs, MWCNTs, and GWCNTs at varying pH values (**a**) (The concentration of HA is 20 mg/L. The concentration of CNTs is 200 mg/L. pH was controlled at 2, 3, 5, 7, 9, 11, and 13. T = 298.15K) and FTIR spectra of effluent from the short-SRT activated sludge system (SRT = 2 d) and three CNTs (**b**).

The effect of solution pH is presented in Fig. 6(a). The equilibrium HA adsorption capacities of the three CNT types first increased, then decreased with pH. The maximum adsorption equilibrium capacities of SWCNTs, MWCNTs, and GWCNTs were 127.0, 101.6, and 39.6 mg·g^−1^ and were attained at pH = 3, 5, and 5, respectively.

## Discussion

In tests of adsorption capacity variation and COD removal rate, the organic matter adsorption rate was high initially owing to the number of effective adsorption sites. Then, the adsorption rate decreased over time due to the reduced number of adsorption sites and the repulsive forces exerted by the adsorbed organic matter against the organics still in the solution. MWCNTs reached their maximum COD adsorption capacity the most quickly, while SWCNTs featured the highest removal rate and TCOD adsorption capacity, albeit over longer periods. These results reveal that the organics removal rate is highly related to the CNT surface area, where greater surface areas produce higher removal rates. GWCNTs showed the lowest COD adsorption capacity and removal rate. Thus, surface area may not be the only factor determining the removal rate, as the rate did not strictly increase with increasing CNT surface area; surface chemical properties are also important. The mechanism involves physical interactions between CNTs and organic matter, such as hydrophilic, hydrophobic, and electrostatic interactions, as well as hydrogen bonds and/or π-π bonds; the size and morphology^29^, polarity^30^, and π-electron properties of the organic matter are also related.

Figure 1(c) shows that the CNT surface area is highly correlated with the removal of PCOD and CCOD. The ratio of SCOD to TCOD increased after the adsorption tests, indicating that CNTs removed more PCOD and CCOD than SCOD. Nevertheless, SCOD still decreased dramatically. As presented in Fig. 1(d), only half of the SCOD remained in the water after adsorption on the three CNTs. The high DOM adsorption performance attained by the CNTs is difficult to achieve via other water treatment techniques.

SUVA increased from 0.55 in the source water to 0.64, 0.74, and 0.77 after adsorption to SWCNTs, MWCNTs, and GWCNTs, respectively, indicating stronger aromaticity and a greater ratio of conjugated double-bond organics after adsorption. The adsorption capacity for DOM containing aromatic rings or conjugated double bonds was stronger for MWCNTs than for GWCNTs, likely due to the increased number of oxygen-containing functional groups. UV_253_/UV_203_ decreased after adsorption to all three CNTs, indicating a lower degree of aromatic ring substitution. Therefore, the adsorption of DOM from the source water to CNTs can reduce potential for disinfection by-product production.

The ratios of TPI-A and HPI increased after the adsorption treatment, and the proportion of HPO-A decreased, revealing that CNTs adsorb HPO-A more readily than they do HPI and TPI-A. The HPO-N ratio rose slightly after adsorption to both SWCNTs and MWCNTs, but decreased sharply after adsorption to GWCNTs. The strong hydrophilicity of MWCNTs, which arises from the larger amounts of surface -OH and -COOH, likely causes this result through reduced interactions with hydrophobic organic compounds^12^. As shown in Fig. 2(c), E_4_/E_6_ decreased dramatically from 3.20 in the source water to 1.67 and 1.75 after adsorption tests using SWCNTs and MWCNTs, respectively, indicating that high molecular weight organics accounted for a larger proportion of the DOM after adsorption. However, the E4/E6 value increased sharply, to 6.00, after treatment by GWCNTs, which have fewer surface functional groups to adsorb low molecular weight organic compounds. This indicates that surface functional groups influence both the adsorption capacity for organics with different molecular weights and the biological stability of the effluent.

In experiments on the effects of temperature, the COD adsorption capacity of the CNTs decreased from 25 to 45 °C, indicating that the adsorption of organics to CNTs is an exothermic process. MWCNT adsorption tests were less affected by temperature than were GWCNT tests, which is consistent with the presence of oxygen functional groups on the MWCNT surface. Previous studies have suggested that surface functional groups such as -OH, -COOH, and -C=O may increase the affinity between CNTs and organic matter via stronger π-π electron interactions, Lewis acid-base interactions, or hydrogen bonding^31,32^. Higher adsorption affinity between CNTs and organic matter would thus reduce the influence of temperature.

In experiments on the effects of CNT concentration (50–300 mg·L^−1^), although the COD removal rates increased with increasing CNT concentration for all three CNTs, the rates of increase slowed. This reveals that some organics cannot be effectively adsorbed by CNTs even when more adsorption sites are added to the solution because physical adsorption fails when the organic molecules are small.

As shown in Fig. 3(f,g), the adsorptive capacities of the CNTs, and especially the SWCNTs and MWCNTs decreased under high solution pH, likely due to the increased number of surface functional groups. pH can change the protonation, solubility, hydrophilicity, and chemical form of organics in solution^7,33^. The protonation of –COOH may have thus promoted the formation of water molecules on the CNT surface, which likely weakened the hydrogen bonds between the CNTs and organic molecules and hindered adsorption^34^. Nevertheless, continuous increases in adsorption when the pH was increased from 2 to 9 reveal that, in certain ranges, higher pH may also promote the adsorption of organics, resulting in stronger electron donor effects between CNTs and organic matter. Overall, the effect of pH on CNT adsorption properties is related primarily to the organic matter ionization constant and the CNT pH_pzc_.

The tests on HA adsorption (where HA was used as a typical organic effluent contaminant) included experiments on adsorption kinetics, isotherm responses, thermodynamics, and the effect of varying pH. The difference in the HA adsorption capacity indicates that larger CNT specific surface areas promote greater HA adsorption capacity, suggesting that van der Waals forces play a major role in the adsorption of HA by CNTs. This result is consistent with previous reports^17^. We argue that the adsorption of HA by CNTs was also affected by surface functional groups and other forces, as the amounts of HA adsorbed on a unit surface area differed between the three CNT types.

The pseudo-second-order model better describes the kinetics of HA adsorption to SWCNTs and MWCNTs. The k_2_ value of the SWCNT pseudo-second-order model was significantly lower than that for the MWCNTs, indicating that the MWCNT HA adsorption rate was larger than that for the SWCNTs. The difference in k_2_ may have been caused by the functional groups on CNT surfaces. Oxygen-containing functional groups such as hydroxyl and carboxyl groups may form hydrogen bonds with the aromatic rings in the HA molecule, promoting the adsorption of HA to CNTs^17^. However, the GWCNT pseudo-first-order model had higher correlation coefficients. The experimental results show that SWCNTs and MWCNTs were both affected by certain chemical bonds in the adsorption process, indicating a more complex chemical adsorption process involving heterogeneous diffusion. However, HA adsorption by GWCNTs was controlled largely by the diffusion step due to differences in the surface functional groups between MWCNTs and GWCNTs.

In Fig. 5, the fitted curve of the internal diffusion kinetic model is not linear; i.e., the adsorption of HA on CNTs did not conform to the internal diffusion model. Therefore, pore diffusion is not the only rate controlling step in the adsorption of HA to CNTs, and adsorption kinetics depends primarily on surface diffusion mechanisms. Table 1b showed that the K_int1_ parameter was significantly larger than K_int2_ for SWCNTs and MWCNTs, while C_1_ was much smaller than C_2_, indicating that, in Stage 1, HA had a high rate of diffusion from the liquid to the CNT surface because the HA Brown movement was strong and the resistance was small; thus, HA spread easily to the solid-liquid interface. A water film was formed due to the presence of hydrophilic functional groups on two of the CNT surfaces, making it difficult for HA to enter the CNT pore. Thus, the HA adsorption rate was dominated by interface layer diffusion. Because the C_1_ of the MWCNTs is 0, the HA adsorption rate was dominated by intrapore diffusion. In Stage 2, with the HA concentration decreasing gradually and the membrane boundary layer becoming thicker, the adsorption rate of HA on SWCNTs and MWCNTs decreased and the adsorption mechanism shifted from surface adsorption to pore absorption. Because C_2_ is positive, the adsorption rate of HA by SWCNTs and MWCNTs was controlled by both internal and external diffusion. The GWCNT C_2_ value is also positive; thus, the HA adsorption rate control mechanism gradually changed from internal diffusion to a combination of internal and external diffusion. Because HA is negatively charged, HA adsorbed on the pores of GWCNTs repulsed unadsorbed HA and inhibited the diffusion of HA into the GWCNT pores. In Stage 3, the adsorption of HA onto the three CNTs was dominated by internal diffusion, with further decreased the HA concentration in the solution and thickened the membrane boundary layer until adsorption equilibrium was reached.

In isotherm studies, the maximum adsorption capacity of the three CNTs decreased as the temperature increased, showing that the adsorption of HA on CNTs is an exothermic process. In addition, most Freundlich model parameters were less than 1, indicating that the HA adsorption isotherms of the three CNTs are highly nonlinear, likely due to the heterogeneous adsorption sites on the CNTs. Graphene sheets in CNTs are rich in π-electron donors, which can form π-π interactions with aromatic ring-containing compounds^35,36^. Oxygen-containing functional groups such as -COOH and -OH enhance the π-π interactions between CNTs and organic matter by increasing the π electron density on the CNT surface^4^. Thus, theoretically, π-π interaction are possible between the CNT surface and HA. Owing to the small number of oxygen-containing functional groups on the GWCNT surface, the π-π interactions on GWCNTs should be weaker than those on MWCNTs. Moreover, both HA and the surfaces of the three CNTs feature a negative charge; therefore, electrostatic repulsion will be produced during the adsorption process. GWCNTs have the highest pH_pzc_ value; hence, this material should be less affected by electrostatic repulsion than the MWCNTs.

In thermodynamic studies, the standard Gibbs free energies, ΔG^θ^, of the three CNTs during the adsorption of HA are less than zero, indicating that the adsorption process is spontaneous. The ΔG^θ^ values of the three CNTs increased with temperature, hindering the spontaneous adsorption of HA on CNTs. The standard enthalpy change, ΔH^θ^, for the adsorption process is less than zero, indicating that the adsorption process is exothermic; the standard entropy change, ΔS^θ^, during the adsorption process is also less than zero, indicating an increase in the orderliness of the reaction system. After the adsorption of HA onto the CNTs, the CNTs showed increasing amounts of negative charge owing to the large amounts of negative charge provided by HA. This increased repulsion between CNTs in the solution and promoted a change in CNT organization from a state of relative union to a more widely-spaced, ordered arrangement. The number of hydrophilic groups on the CNT surfaces also increased as more HA was adsorbed. This led to a larger number of water molecules around the CNTs interacting with the CNTs through hydrogen bonds to form a large molecule-network structure, which led to an increase in order via the adsorption process^37^.

From Fig. 6(a), the maximum adsorption equilibrium capacities of SWCNTs, MWCNTs, and GWCNTs were attained at pH = 3, 5 and 5, respectively. This pattern is related primarily to the pH_pzc_ values of the three CNTs and the degree of HA electrolysis. When organic matter dissociates due to a change in pH, the solubility of the organic matter is greatly increased while the hydrophobic interactions with CNTs are reduced. The ability of the functional groups in the organic matter to provide protons that form hydrogen bonds with the CNTs is also weakened because of functional group ionization. When the charges carried by the organic functional groups are the same as those on the CNT surface, electrostatic repulsion is also enhanced. Therefore, the adsorption of HA on CNTs was inhibited as the pH of the reaction solution gradually increased. The three CNTs displayed especially sharp decreases in HA adsorption capacity at pH greater than 6. Previous studies reached a similar conclusion, finding that oxygen-containing functional groups such as -COOH and -OH dissociated and the electron donating ability of organic compounds increased under increasing pH, strengthening the π-π forces between the CNTs and organic matter^38^. However, at high pH, the π-π effect did not greatly affect HA adsorption according to our experimental results. Because the pH_pzc_ values of the three CNTs are less than 7, solution pH values closer to the CNT pH_pzc_ value should decrease repulsion between HA and the CNTs and increase the strength of HA adsorption on the CNTs. Thus, SWCNTs, MWCNTs, and GWCNTs attained their maximum adsorption capacities when the solution pH was close to their respective pH_pzc_ values.

Generally, no large differences were observed between the SWCNT and MWCNT adsorption characteristics, although MWCNTs have been reported to possess a more layered structure. HA has a macromolecular structure, which impedes its capture in the interlayer spaces in MWCNTs. SWCNTs have better adsorption properties than MWCNT owing to the smaller diameter distribution along with higher uniformity of SWCNT. In contrast, the adsorption of natural organic material by GWCNTs was significantly different from that by MWCNTs due to the lack of oxygen-containing surface functional groups on the GWCNTs. Nevertheless, all three CNTs can remove more than 40% of the SCOD and TCOD, induce higher organic matter stability in the effluent, and decrease the potential for disinfection by-product production, demonstrating their superior potential for the advanced treatment of effluent from short SRT activated sludge systems.

## Methods

### Adsorbents

The adsorbents used in this study included one SWCNT, one MWCNT, and one GWCNT. All CNTs were purchased from Chengdu Organic Chemicals Co. Ltd. (China). These CNTs (purity > 99.0%) were used for the experiment as received. The surface areas and total pore volumes of the three CNTs were measured using N_2_ adsorption/desorption isotherms performed at 77 K with a Tristar 3020 II and the BET method. The three CNT samples were dispersed in Milli-Q water and sonicated for 15 min before the tests. The measured surface areas of the SWCNT, MWCNT, and GWCNT substrates are 210.5, 142.3, and 97.7 m^2^·g^−1^, respectively; the total pore volumes are 0.29, 0.23, and 0.13 cm3·g-1, while the mean pore diameters are 5.45, 6.47, and 5.38, respectively. The zeta potentials (pH_pzc)_ of SWCNTs, MWCNTs, and GWCNTs are 3.9, 5.3, and 6.5, indicating that CNTs are negatively charged; more oxygen-containing functional groups on the surface may lead to lower pH_pzc_ and more negative charge.

Basically, CNT surfaces may contain more or fewer functional groups, such as -OH, -COOH and -C=O, which may cause significant differences in adsorptive properties. As shown in Fig. 6(b), Fourier transform infrared (FTIR) spectra of effluent from the short-SRT activated sludge system (SRT = 2d) and three CNTs exhibits the functional groups in the source water and on the surface of CNTs. Obviously, the DOM in the source water contains lots of functional groups, such as carboxyl, hydroxyl, and amino groups. The absorbance bands at 3,450 cm^−1^ indicate hydroxyl groups, and GWCNT has the lowest surface hydroxyl content of the three CNTs. The strong absorbance bands at 1,650–1,750 cm^−1^ denote carboxyl groups; similarly, GWCNT has the lowest surface carboxyl content. The bands at ~1,450 cm^−1^ may be attributed to C-O, while the bands at 1,000 cm^−1^ represent C-C and C-H. The hydroxyl and carboxyl group contents were determined from the FTIR spectra, and the resulting data are listed in Table 2. The GWCNT material has smaller surface area, mean pore diameter, and total pore volume and fewer surface functional groups than does the MWCNT, leading to strong hydrophobicity.

**Table 2:** Properties of CNTs used in the experiments.

Carbon type	SA_BET_^a^ m^2^·g^−1^	V_TP_^b^ cm^3^·g^−1^	D_mp_^c^ nm	Surface acidic functional groups (mmol·g^−1^)			pH_pzc_^d^
				Hydroxyl	Carboxyl	Total	
SWCNTs	210.5	0.29	5.45	0.025	0.038	0.063	3.9
MWCNTs	142.3	0.23	6.47	0.013	0.134	0.147	5.3
GWCNTs	97.7	0.13	5.38	0.003	0.005	0.008	6.5

^a^Specific surface area calculated with the BET model.

^b^Total pore volume calculated from single point adsorption P/P_0_ = 0.99.

^c^Mean pore diameter obtained using the BET model.

^d^Point of zero charge (PZC) obtained via batch adsorption tests.

### Source water and target organic contaminant

Effluent from the short-SRT activated sludge system (SRT = 2 d) for domestic sewage was selected as the target source water for each batch experiment with 60–80 mg·L^−1^ COD. Humic acid was selected as the target organic contaminant and was used directly without any other treatment.

### Other materials

NaCl, NaOH, HCl, H_2_SO_4_, potassium bromide (KBr), and other analytical grade reagents were purchased from Sinopharm Chemical Reagent Co., Ltd. In all tests, the solution pH was adjusted using 1M HCl and 1M NaOH.

### Experimental instrument and equipment

COD was measured using a COD rapid detector of LH-3B. Centrifugation was conducted on a SiGMA^@^3K15 centrifuge. An electronic balance of GR202 was used to measure the weight of CNTs and reagents. FTIR spectra were collected on a Bruker TENSOR 27. The HA concentrations were measured using a fluorescence spectrophotometer (F9700S). Milli-Q Integral Water Purification System was used to produce ultrapure water. The reagent dosing was performed using the pipettes of Gilson, Inc. PHS-3C was used to determine the pH of the solutions. SHA-C Water Bath Shaker was used for oscillation at constant temperature.

### Measurement of substances involved in the experiments

The CNT organic functional groups were detected via FTIR using the KBr pressed-disk technique. The sample scanning range was 400‒4,000 cm^−1^ with a resolution of 4 cm^−1^ in transmission mode at room temperature.

The CNT point of zero charge (PZC) values were obtained via batch adsorption tests. For each type of CNT, 5 mg of adsorbent was shaken in a polypropylene tube with 25 mL of 0.02 M NaCl at background pH (pH_i_) values ranging between 2 and 11. After shaking for 6 h at 25 °C, the final pH (pH_f_) was measured. Plots of ΔpH (pH_i_ –pH_f_) versus pH can be used to find the PZC, which is located where the curve meets the x-axis.

COD fractionation is generally based on a single cut size, namely the routine filtration size (1.5 or 0.45 μm) used to differentiate particulate, colloidal, and soluble COD components. PCOD was defined herein as the difference between TCOD and COD after filtration with a 1.5 μm membrane; i.e., PCOD = TCOD−COD_1.5_. SCOD refers to the COD remaining after filtration with a 0.45 μm membrane. As such, CCOD = COD_1.5_−SCOD^39^.

Specific UV Absorbance (SUVA) and UV253/UV203 were measured using a fluorescence spectrophotometer in order to indicate the potential for the production of disinfection by-products by DOM in the water. Effluent hydrophilicity analysis was conducted using XAD-8 and XAD-4 resin columns in series to measure HPO-A (the acid hydrophobic fraction), HPO-N (the non-acid hydrophobic fraction), TPI-A (the acid transphilic fraction), and HPI (the hydrophilicity), which can then be used to explore SCOD composition and characteristics. HA was detected at a wavelength of 254 nm on a fluorescence spectrophotometer.

### Testing for COD adsorption in short SRT wastewater treatment effluent

Adsorption tests for effluent COD from the short SRT wastewater treatment process, and for typical organic contaminants in the effluent SCOD, were carried out on SWCNT, MWCNT, and GWCNT materials in the batch mode. To determine variations in COD adsorption with time, 30 mg of the target carbon nanotube was mixed with 150 mL of the short SRT wastewater treatment effluent in a 250 mL centrifuge bottle. The solutions were controlled at 25 °C in a water bath and stirred at 250 rpm. At 0 min, 15 min, 30 min, 45 min, 1 h, 1.5 h, 2 h, 3 h, 4 h, and 6 h, 10 mL of solution was withdrawn and centrifuged at 4,000 rpm for 5 min to separate the carbon nanotubes in order to measure COD.

To explore COD adsorption at different temperatures, 5 mg of the given carbon nanotube was mixed with 25 mL of source water in a 30 mL centrifuge bottle. The solutions were controlled at 25, 35, and 45 °C in a water bath and stirred at 250 rpm. The solution was withdrawn after 6 h of stirring and centrifuged at 4,000 rpm for 5 min to separate the carbon nanotube in order to measure COD. The tests to investigate COD adsorption under different adsorbent concentrations and pH values were quite similar, except that the solutions were mixed with carbon nanotubes of varying concentrations and pH was controlled at 2, 3, 5, 7, 9, 11, and 13.

### Testing for typical organic contaminants in SCOD in ultra-short SRT wastewater treatment effluent

An HA stock solution was prepared in 0.02 mol·L^−1^ NaCl. Except for the adsorption tests at different pH values, all experiments were conducted at pH 5.5 to safeguard the molecular structure of HA. For the kinetic experiments, 30 mg each of carbon nanotubes and HA were mixed with 150 mL of 0.02 mol·L^−1^ NaCl in a 250 mL centrifuge bottle. The solution was controlled at 25 °C in a water bath and stirred at 250 rpm. At 0 min, 15 min, 30 min, 45 min, 1 h, 1.5 h, 2 h, 3 h, 4 h, and 6 h, 10 mL solution was withdrawn and filtered using a 0.45 μm membrane before HA concentration analysis.

For the isotherm experiments, 5 mg each of carbon nanotubes and HA were mixed with 25 mL of 0.02 mol·L^−1^ NaCl in a 30 mL centrifuge bottle. The solution was controlled at 25, 35, and 45 °C in a water bath and stirred at 250 rpm. Tests for HA adsorption at different concentrations and pH values were similar, except that varying HA concentrations were used and pH was controlled at 2, 3, 5, 7, 9, 11, and 13. All solutions were withdrawn after 6 h of stirring and filtered using a 0.45- μm membrane before measuring the HA concentration.
